# Production Strategies and Applications of Microbial Single Cell Oils

**DOI:** 10.3389/fmicb.2016.01539

**Published:** 2016-10-05

**Authors:** Katrin Ochsenreither, Claudia Glück, Timo Stressler, Lutz Fischer, Christoph Syldatk

**Affiliations:** ^1^Technical Biology, Institute of Process Engineering in Life Sciences, Karlsruhe Institute of TechnologyKarlsruhe, Germany; ^2^Biotechnology and Enzyme Science, Institute of Food Science and Biotechnology, University of HohenheimStuttgart, Germany

**Keywords:** single cell oil, solid-state fermentation, submerged fermentation, downstream processing, food application

## Abstract

Polyunsaturated fatty acids (PUFAs) of the ω-3 and ω-6 class (e.g., α-linolenic acid, linoleic acid) are essential for maintaining biofunctions in mammalians like humans. Due to the fact that humans cannot synthesize these essential fatty acids, they must be taken up from different food sources. Classical sources for these fatty acids are porcine liver and fish oil. However, microbial lipids or single cell oils, produced by oleaginous microorganisms such as algae, fungi and bacteria, are a promising source as well. These single cell oils can be used for many valuable chemicals with applications not only for nutrition but also for fuels and are therefore an ideal basis for a bio-based economy. A crucial point for the establishment of microbial lipids utilization is the cost-effective production and purification of fuels or products of higher value. The fermentative production can be realized by submerged (SmF) or solid state fermentation (SSF). The yield and the composition of the obtained microbial lipids depend on the type of fermentation and the particular conditions (e.g., medium, pH-value, temperature, aeration, nitrogen source). From an economical point of view, waste or by-product streams can be used as cheap and renewable carbon and nitrogen sources. In general, downstream processing costs are one of the major obstacles to be solved for full economic efficiency of microbial lipids. For the extraction of lipids from microbial biomass cell disruption is most important, because efficiency of cell disruption directly influences subsequent downstream operations and overall extraction efficiencies. A multitude of cell disruption and lipid extraction methods are available, conventional as well as newly emerging methods, which will be described and discussed in terms of large scale applicability, their potential in a modern biorefinery and their influence on product quality. Furthermore, an overview is given about applications of microbial lipids or derived fatty acids with emphasis on food applications.

## Introduction

Single cell oils (SCOs) are intracellular storage lipids comprising of triacyglycerols (TAGs). SCOs are produced by oleaginous microorganisms which are able to accumulate between 20% and up to 80% lipid per dry biomass in the stationary growth phase under nutrient limitations, e.g., nitrogen or phosphor, with simultaneous excess of carbon source. Depending on the oleaginous microorganism including bacterial, yeast, microalgae or fungal species, fatty acid profile of SCOs can vary making them highly suitable for diverse industrial applications.

Considering the foreseeable depletion of crude oil, the highly controversial “food-or-fuel” discussion about using plant oils for biodiesel production, overfishing of the oceans and the urgent need for the reduction of greenhouse gas emissions, microbial SCOs seems to be intriguing substitutes for crude, plant, and fish oil. Furthermore, microbial lipid production is independent from season, climate, and location, can be realized using a wide range of carbon source, e.g., waste streams from food industry or renewable carbon sources, in case of microalgae even from CO_2_, does not use arable land, results in high yields and can be accomplished with genetically modified organisms changing fatty acid composition and enhancing yields. Whereas the production of very long polyunsaturated fatty acids, i.e., docosahexaenoic acid (DHA; 22:6, ω-3) and arachidonic acid (ARA; 20:4, ω-6), are commercialized using the oleaginous fungus *Mortierella alpina* and different oleaginous microalgae (for an overview see Ratledge, 2004), the production of biodiesel from SCO is still not economically competitive.

The ability of (eukaryotic) oleaginous organisms to accumulate large amount of lipids is not accounted to a difference in fatty acid biosynthesis compared to non-oleaginous species. However, a continuous supply of acetyl-CoA and NADPH for the fatty acid production by a reversed β-oxidation has to be assured under nutrient limited but carbon excess conditions. The continuous production of acetyl-CoA in oleaginous microorganisms is achieved by a cascade of enzyme reactions triggered by a nutrient limitation (in biotechnology, usually a nitrogen limitation is used) leading essentially to a citrate accumulation in the mitochondria. A unique feature of oleaginous organisms is the AMP-dependency of isocitrate dehydrogenase, an enzyme of the TCA cycle catalyzing the oxidative decarboxylation of isocitrate. In case of a nitrogen limitation, the activity of AMP deaminase, catalyzing the cleavage of AMP to IMP and ammonia, is increased considerably due to the nitrogen limitation leading to low AMP levels inside the mitochondria. As a consequence, isocitrate is not further metabolized and converted to citrate by the enzyme aconitase. Citrate is transported into the cytosol and cleaved by the enzyme ATP:citrate lyase to acetyl-CoA and oxaloacetate leading eventually to the continuous supply of acetyl-CoA for fatty acid synthesis. ATP:citrate lyase was found so far in all reported oleaginous microorganisms, however, in some non-oleaginous organisms the enzyme is also present (Botham and Ratledge, 1979; Boulton and Ratledge, 1981a,b; Evans and Ratledge, 1984, 1985; Wynn et al., 2001; Ratledge, 2002).

**Figure 1:**
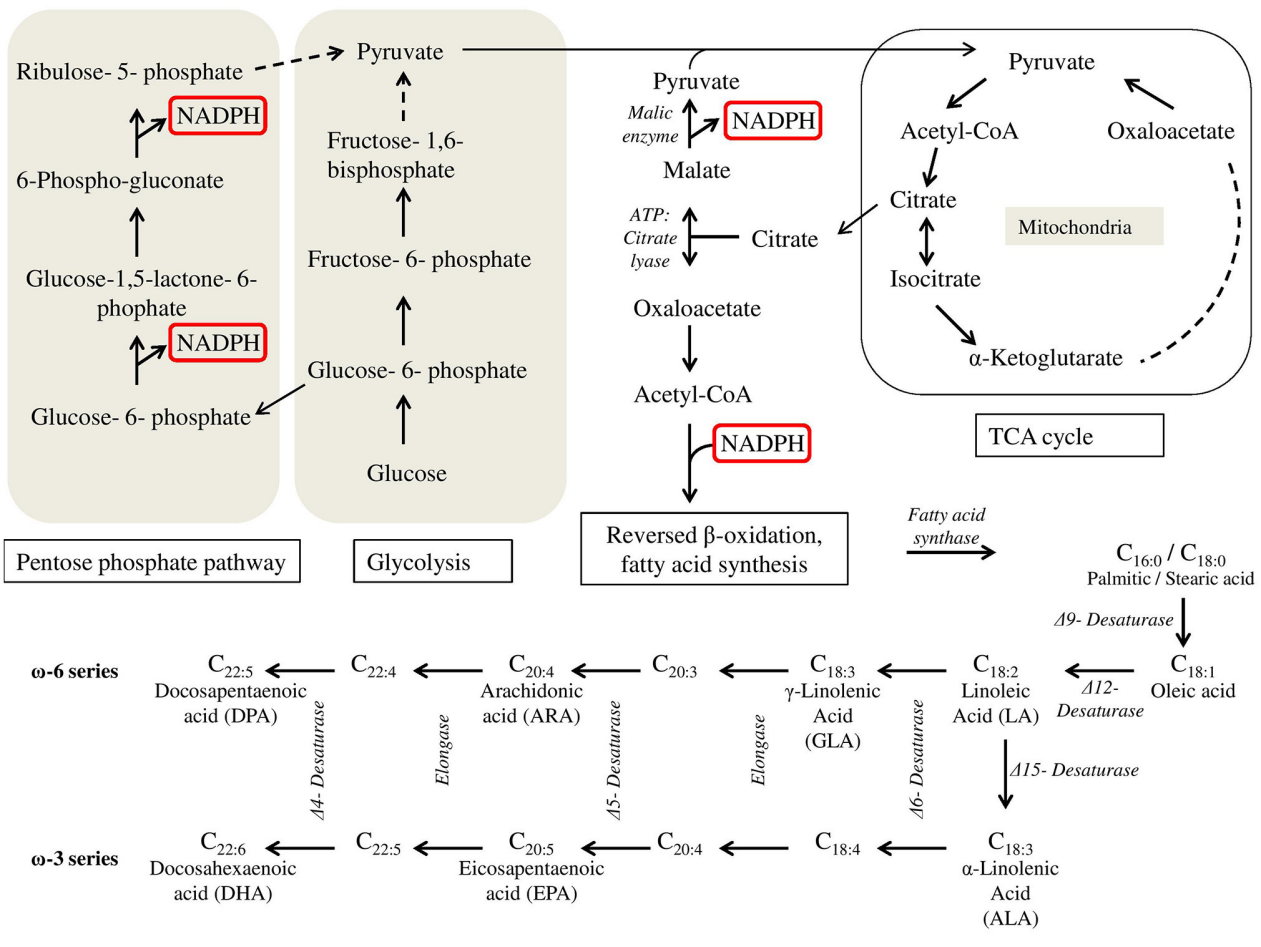
**Biosynthesis of fatty acids under SCO producing conditions in oleaginous eukaryotic microorganisms (adapted and modified after Chemler et al., 2006; Tang et al., 2015)**.

Besides acetyl-CoA a lot of reducing power in form of NADPH is necessary for the production of fatty acids, i.e., 16 moles of NADPH for the synthesis of stearic acid (C18). Although not finally clarified yet, malic enzyme is discussed to be mainly responsible for NADPH supply. Malic enzyme catalyzes the decarboxylation of malate (resulting from oxaloacetate) to pyruvate which is transported into the mitochondria. However, as malic enzyme activity has been reported not to be involved in NADPH regeneration in some oleaginous organisms, e.g., *Yarrowia lipolytica* (Zhang et al., 2013), alternative routes may also be responsible. Additionally, NADPH regeneration via the pentose phosphate pathway is also an option (Tang et al., 2015; Zhao et al., 2015). An overview about the biosynthesis is given in Figure 1.

The described fatty acid biosynthesis ends in almost every organism with the formation of palmitic (16:0) or stearic (18:0) acid. For the production of the especially desired polyunsaturated fatty acids a subsequent series of elongation and desaturation by elongases and desaturases, respectively, is necessary. Therefore, the potential of amount and type of produced PUFA is dependent on the genes of elongases and desaturases present in the genome of the respective oleaginous organism. However, only algae and fungi seem to have the ability to produce SCO containing more than 20% PUFAs making them commercially interesting.

During the last years and decades many studies and reviews dealing with SCO production have occurred. Since the commercialization of SCO production besides the mentioned PUFA production is still uneconomical, more and more researchers are focusing now on combined approaches of genetic engineering to enhance yields and productivity and the usage of low-cost substrates. However, a holistic assessment of the processes including downstream processing is often missing which is orientated on industrial scale and subsequent application of the oil. Therefore, this review aims to give an overview on processes and downstream processing methods suitable for large and industrial scale considering the limitations occurring by the final application of the product.

## Microorganisms for SCO production

**Figure 2:**
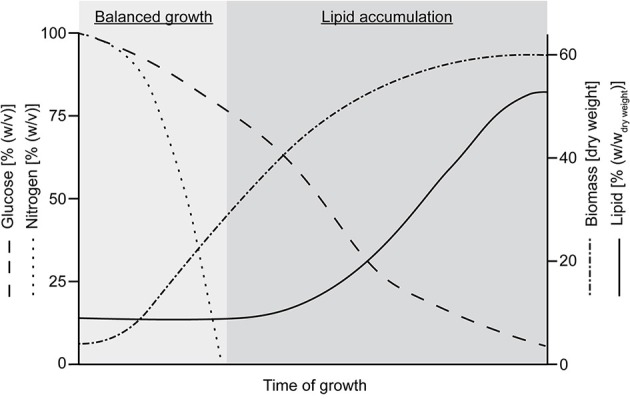
**Typical course of lipid accumulation by oleaginous microorganisms (modified according to Wynn and Ratledge, 2005)**.

Lipids and oil are produced by all living macro- and microorganisms for essential structural and functional roles such as the formation of permeable membranes of cells and organelles in the form of a lipid bilayer (Dowhan and Bogdanov, 2013). However, only a relatively small number of microorganisms are able to accumulate amounts of cellular lipids over 20 or even up to 80% of their cell mass as a reserve storage material. These are termed as oleaginous microorganisms (Ratledge, 2004). The microbial production of SCO offers several advantages compared to the use of animal or plant sources. The cultivation of microorganisms is independent from geographic or climatic constraints, has short producing periods and several substrates, including industrial wastes, can be used (Ward and Singh, 2005b; Li Q. et al., 2008). The main producers of lipids are fungi, yeasts, and algae, while bacteria are bad producers (Wynn and Ratledge, 2005; Li Y. et al., 2008; Bellou et al., 2016). The lipid accumulation as a reserve storage is triggered by an excess of carbon source and one limiting nutrient, usually nitrogen. Under these conditions the carbon flux is directly channeled toward lipid synthesis and discrete oil droplets consisting of triacylglycerols are formed within the cells (Ratledge, 2004; Wynn and Ratledge, 2005). The typical course of lipid accumulation by oleaginous microorganisms is shown in Figure 2.

The first phase of balanced growth, where all nutrients are in excess is characterized by the production of biomass and the consumption of the carbon and nitrogen source. If the nitrogen is exhausted, the biomass production is reduced and the accumulation of lipid starts (Wynn and Ratledge, 2005). In contrast, non-oleaginous microorganisms would either stop cell division or accumulate polysaccharides, including glycogens and various mannans, glucans etc. (Ratledge, 2004). An overview on the microorganisms used for SCO production and the range of produced cellular lipids in their dried biomass is given in Table 1.

**Table 1:** **Overview of the genera used for the production of single cell oil (SCO) and amounts of cellular lipids accumulated per dry weight**.

**Kingdom**	**Division**	**Order**	**Genus**	**SCO [% (w/w_DW_)]**	**References**
Chromalveolata	Heterokontophyta	Labyrinthuales	*Aurantiochytrium*	65	Huang et al., 2012
			*Schizochytrium*	49–67	Chang et al., 2013; Ling et al., 2015
		Phyitales	*Pythium*	76	Cheng et al., 1999
Fungi	Ascomycota	Eurotiales	*Aspergillus*	18	Lin et al., 2010
		Saccharaomycetales	*Candida*	2–27	Chatzifragkou et al., 2011
			*Yarrowia*	7–43	Papanikolaou and Aggelis, 2002; Chatzifragkou et al., 2011
			*Zygosaccharomyces*	13	Chatzifragkou et al., 2011
	Basidiomycota	Sporidiales	*Rhodotorula*	22–52	Zhao et al., 2010; Chatzifragkou et al., 2011
		Sporidiobolales	*Sporobolomyces*	30–50	Matsui et al., 2011
		Tremellales	*Cryptococcus*	33–78	El-Fadaly et al., 2009; Chi et al., 2011
		Ustilaginales	*Rhodosporidium*	33	Matsakas et al., 2015
	Zygomycota	Mucorales	*Cunninghamella*	21–78	Gema et al., 2002; Fakas et al., 2009b; Chatzifragkou et al., 2010, 2011
			*Mucor*	18	Chatzifragkou et al., 2011
			*Thamnidium*	43	Chatzifragkou et al., 2011
			*Zygorhynchus*	42	Chatzifragkou et al., 2011
		Mortierellas	*Mortierella*	5–74	Bajpai et al., 1991; Fakas et al., 2009b; Chatzifragkou et al., 2010; Economou et al., 2011a; Gao et al., 2013; Stressler et al., 2013; Zeng et al., 2013

High amounts of cellular lipids are produced by microorganisms belonging to the genera *Cryptococcus, Cunninghamella*, and *Mortierella*. The genus *Mortierella* is capable to produce SCO with a unique composition, containing high amounts of PUFAs (Asadi et al., 2015). *M. alpina* is used in an industrial process for the production of arachidonic acid (ARA, 20:4, ω-6) for food supplementation by DSM (Béligon et al., 2016).

## Processes for the microbial production of SCO

The microbial production of SCO can be either conducted as submerged (SmF) or solid state fermentation (SSF).

### SmF for SCO production

Table 2 summarizes culture conditions for SCO production by SmF and the cellular lipid contents obtained. The amount of lipids accumulated mainly depends on the mode of cultivation, the carbon and nitrogen source, pH and temperature.

**Table 2:** **SmF for the production of SCO: Species, lipid content, dry weight of biomass (DW), and cultivation conditions**.

**Species**	**SCO [% (w/w_DW_)]**	**DW [g L^−1^]**	**Cultivation mode**	**Carbon source**	**Nitrogen source**	**pH [-]**	**Temperature [°C]**	**Duration**	**SCO Composition**	**References**
*Aspergillus oryzae* A-4	18.2	4.3	Batch, flask	Glucose + cellulose	Yeast extract + (NH_4_)_2_SO_4_	5.5	30°C	360 h	27.7% PUFA	Lin et al., 2010
*Aurantiochytrium limacinum* SR21	65.2	61.7	Fedbatch, STR	Glycerol	Yeast extract + peptone	6.8–7.2	22°C	192 h	66.3–87.9% PUFA	Huang et al., 2012
*Candida pulcherrima* LFMB 1	1.5	7.3	Batch, flask	Waste gycerol	Yeast extract + (NH_4_)_2_SO_4_	5–6	28°C	63 h	16.3% PUFA	Chatzifragkou et al., 2011
*Candida boidinii* ATTC 32195	27.2	1.3	Batch, flask	Waste gycerol	Yeast extract + (NH_4_)_2_SO_4_	5–6	28°C	111 h	15.6% PUFA	Chatzifragkou et al., 2011
*Candida curvata* NRRL-Y 1511	6.5	7.9	Batch, flask	Waste gycerol	Yeast extract + (NH_4_)_2_SO_4_	5–6	28°C	137 h	12.0% PUFA	Chatzifragkou et al., 2011
*Candida oleophila* ATCC 20177	15.3	9.4	Batch, flask	Waste gycerol	Yeast extract + (NH_4_)_2_SO_4_	5–6	28°C	91 h	11.4% PUFA	Chatzifragkou et al., 2011
*Cryptococcus curvatus* NRRL-Y 1511	78	1.8	Batch, flask	Tomato peels	NaNO_3_	5.8–6.0	28°C	72 h	–	El-Fadaly et al., 2009
	77	1.3	Batch, flask	Glucose	Rice bran	4.2–6.0	28°C	72 h	–	El-Fadaly et al., 2009
	73	2.2	Batch, flask	Potato peels	NaNO_3_	4.6–6.0	28°C	72 h	–	El-Fadaly et al., 2009
	67	1.5	Batch, flask	Glucose	Protelan	4.5–6.0	28°C	72 h	–	El-Fadaly et al., 2009
	64	2.5	Batch, flask	Sugar cane molasses	NaNO_3_	4.9–6.0	28°C	72 h	–	El-Fadaly et al., 2009
	57	2.8	Batch, flask	Glucose	Corn gluten	4.7–6.0	28°C	72 h	–	El-Fadaly et al., 2009
	56	3.2	Batch, flask	Sugar beet molasses	NaNO_3_	6.0–7.2	28°C	72 h	–	El-Fadaly et al., 2009
	42	2.5	Batch, flask	Glucose	Corn steep liquor	5.3–6.0	28°C	72 h	–	El-Fadaly et al., 2009
	34	3.5	Batch, flask	Glucose	NaNO_3_ (control)	5.5–6.0	28°C	72 h	–	El-Fadaly et al., 2009
	33	3.6	Batch, flask	Glucose (control)	NaNO_3_	5.3–6.0	28°C	72 h	-	El-Fadaly et al., 2009
*Cryptococcus curvatus* ATCC 20509	75	168	Fedbatch, STR	Hydrogen prduction effluent + acetic acid	NH_4_Cl	7	30°C	192 h	15.6% PUFA	Chi et al., 2011
*Cunninghamella echinulata* ATHUM 4411	57.7	7.8	Batch, flask	Xylose	Yeast extract + (NH_4_)_2_SO_4_	5.2–6.0	28°C	192 h	6.6% GLA	Fakas et al., 2009b
	46.6	5.5	Batch, flask	Glucose	Yeast extract	6.0	28°C	193 h	14% GLA	Gema et al., 2002
	46	15	Batch, flask	Glucose	Yeast extract + (NH_4_)_2_SO_4_	5.2–6.2	28°C	360 h^a^	–	Fakas et al., 2009b
	36.3	4.3	Batch, flask	Waste gycerol	Yeast extract + (NH_4_)_2_SO_4_	5.0–6.0	28°C	135 h	26.1% PUFA	Chatzifragkou et al., 2011
	32	12.1	Batch, flask	Molasse	Yeast extract + (NH_4_)_2_SO_4_	5.2–6	28°C	356 h	27.4 PUFA	Chatzifragkou et al., 2010
	30	12.9	Batch, flask	Commercial glucose	Yeast extract + (NH_4_)_2_SO_4_	5.2–6	28°C	309 h	35.8% PUFA	Chatzifragkou et al., 2010
	25.6	7.8	Batch, flask	Waste gycerol	Yeast extract + (NH_4_)_2_SO_4_	5.2–6.1	28°C	340 h	9.5% GLA	Chatzifragkou et al., 2011
	21	16.7	Batch, flask	Commercial fructose	Yeast extract + (NH_4_)_2_SO_4_	5.2–6.0	28°C	405 h	30.6% PUFA	Chatzifragkou et al., 2010
*Mortierella alpina*	49	–	Batch, STR	Glucose	Yeast extract	–	20°C	13 days	38% ARA	Stressler et al., 2013
	27.3	16.5	Batch, flask	Glucose	Yeast extract	–	20°C	8–10 days	63% PUFA, 38% ARA	Stressler et al., 2013
*Mortierella alpine* ATCC 32222	22.3	15.15	Batch, flask	Glucose	Yeast extract + KNO_3_	–	11°C	600 h	48.1% PUFA	Bajpai et al., 1991
	17.8	17.5	Batch, flask	Glucose	Yeast extract + KNO_3_	–	25°C	360 h	63.9% PUFA	Bajpai et al., 1991
	13.2	6.39	Batch, flask	Glucose	Polypeptone + yeast extract + malt extract	–	25°C	360 h	21.0% PUFA	Bajpai et al., 1991
	10.4	9.27	Batch, flask	Glucose	Yeast extract	–	25°C	360 h	63.2% PUFA	Bajpai et al., 1991
*Mortierella isabellina* ATHUM 2935	74	13.2	Batch, flask	Commercial glucose	Yeast extract + (NH_4_)_2_SO_4_	5.2–6	28°C	237 h	8.5% PUFA	Chatzifragkou et al., 2010
	72	17.8	Batch, STR	Commercial glucose	Yeast extract + (NH_4_)_2_SO_4_	6.0	28°C	160 h	–	Chatzifragkou et al., 2010
	65.5	8.7	Batch, flask	Xylose	Yeast extract + (NH_4_)_2_SO_4_	5.2–6.3	28°C	216 h	3.9% GLA	Fakas et al., 2009b
	64.3	6^a^	Batch, flask	Rice hull hydrolysate	Rice hull hydrolysate	6.0–6.4	28°C	310 h	19.5% PUFA	Economou et al., 2011b
	61	12.1	Batch, flask	Commercial fructose	Yeast extract + (NH_4_)_2_SO_4_	5.2–6	28°C	405 h	12.1% PUFA	Chatzifragkou et al., 2010
	54	9.5	Batch, flask	Molasse	Yeast extract + (NH_4_)_2_SO_4_	5.2–6	28°C	150 h	18.7% PUFA	Chatzifragkou et al., 2010
	53.2	6.2	Batch, flask	Waste gycerol	Yeast extract + (NH_4_)_2_SO_4_	5.2–6.4	28°C	264 h	1.9% GLA	Fakas et al., 2009b
	44.6	27	Batch, flask	Glucose	Yeast extract + (NH_4_)_2_SO_4_	5.2–6.5	28°C	360 h^a^	–	Fakas et al., 2009b
*Mortierella isabellina* MUCL 15102	33.2	5.6	Batch, flask	Waste gycerol	Yeast extract + (NH_4_)_2_SO_4_	5–6	28°C	186 h	18.9% PUFA	Chatzifragkou et al., 2011
*Mortierella isabellina* NRRL 1757	66.7	6.0	Batch, flask	Xylose	Yeast extract + (NH_4_)_2_SO_4_	5.5	28°C	360 h	17.8% PUFA	Zeng et al., 2013
	66.5	8.7	Batch, flask	Glucose	Yeast extract + (NH_4_)_2_SO_4_	5.5	28°C	360 h	14.3% PUFA	Zeng et al., 2013
	62.5	6.1	Batch, flask	Fructose	Yeast extract + (NH_4_)_2_SO_4_	5.5	28°C	360 h	14.0% PUFA	Zeng et al., 2013
	51.2	9.4	Batch, flask	Mannose	Yeast extract + (NH_4_)_2_SO_4_	5.5	28°C	360 h	13.7% PUFA	Zeng et al., 2013
	49.1	8.2	Batch, flask	Galactose	Yeast extract + (NH_4_)_2_SO_4_	5.5	28°C	360 h	15.0% PUFA	Zeng et al., 2013
	48.2	5.8	Batch, flask	Arabinose	Yeast extract + (NH_4_)_2_SO_4_	5.5	28°C	360 h	22.1% PUFA	Zeng et al., 2013
	40.9	5.8	Batch, flask	Cellobiose	Yeast extract + (NH_4_)_2_SO_4_	5.5	28°C	360 h	17.1% PUFA	Zeng et al., 2013
	4.8	0.28	Batch, flask	CMC	Yeast extract + (NH_4_)_2_SO_4_	5.5	28°C	360 h	43.3% PUFA	Zeng et al., 2013
	30.2	3.4	Batch, flask	Ribose	Yeast extract + (NH_4_)_2_SO_4_	5.5	28°C	360 h	19.9% PUFA	Zeng et al., 2013
	16.9	3.7	Batch, flask	Sucrose	Yeast extract + (NH_4_)_2_SO_4_	5.5	28°C	360 h	22.0% PUFA	Zeng et al., 2013
	64.2	28.8	Batch, flask	Xylose	Yeast extract	6.0	28°C	540 h	13.4% PUFA	Gao et al., 2013
	34	12.6	Batch, flask	Straw hydrolysate	Yeast extract + (NH_4_)_2_SO_5_	5.5	28°C	360 h	11.6% PUFA	Zeng et al., 2013
*Mortierella ramanniana* MUCL 9235	37.1	7.3	Batch, flask	Waste gycerol	Yeast extract + (NH_4_)_2_SO_4_	5.0–6.0	28°C	216 h	22.4% PUFA	Chatzifragkou et al., 2011
*Mucor* sp. LGAM 365	18.1	5.3	Batch, flask	Waste gycerol	Yeast extract + (NH_4_)_2_SO_4_	5.0–6.0	28°C	237 h	31.8% PUFA	Chatzifragkou et al., 2011
*Pythium irregular* ATCC 10951	76	35	Batch, flask	Glucose + Soybean oil	(NH_4_)_2_SO_4_	6.0	18°C	10 days	2.0% EPA, 1.0% ARA	Cheng et al., 1999
*Rhodosporidium toruloides* CCT 0783	33	40 ^a^	Batch, flask	Sorghum stalks		–	30°C	250 h		Matsakas et al., 2015
*Rhodotorula mucilaginosa* TJY15a	52.2	19.5	Fedbatch, STR	Artichoke tuber extract hydrolysate	Yeast extract + (NH_4_)_2_SO_4_	6.0	30°C	108 h	11.3% PUFA	Zhao et al., 2010
	48.8	14.8	Batch, flask	Inulin hydrolysate	Yeast extract + (NH_4_)_2_SO_4_	6.0	28°C	72 h		Zhao et al., 2010
	48.6	14.5	Batch, flask	Artichoke tuber extract hydrolysate	Yeast extract + (NH_4_)_2_SO_4_	6.0	28°C	72 h		Zhao et al., 2010
*Rhodotorula* sp. LFMB 22	22	8	Batch, flask	Waste gycerol	Yeast extract + (NH_4_)_2_SO_4_	5.0–6.0	28°C	168 h	12.4% PUFA	Chatzifragkou et al., 2011
*Schizochytrium* sp. LU310	67.3	60.8	Batch, flask	Glucose	Corn steep powder + MSG	6.5	28°C	120 h	41% DHA	Ling et al., 2015
	54.6	46.8	Batch, unbaffeld flasks	Glucose	Corn steep powder + MSG	6.5	28°C	120 h	33% DHA	Ling et al., 2015
*Schizochytrium* sp. S31	49.1	40 ^a^	Batch, flask	Glycerol	Yeast extract + (NH_4_)_2_SO_4_	6.8	28°C	80 h		Chang et al., 2013
*Sporobolomyces carnicolor* O33	50	3.3	Batch, flask	Glucose	Urea	5.6	–	10 h		Matsui et al., 2011
	30	1.6	Batch, flask	Glucose	(NH_4_)_2_SO_4_	5.6	–	7 h		Matsui et al., 2011
*Thamnidium elegans* CCF 1465	42.6	6.8	Batch, flask	Waste gycerol	Yeast extract + (NH_4_)_2_SO_4_	5.0–6.0	28°C	271 h	15.7% PUFA	Chatzifragkou et al., 2011
*Yarrowia lipolytica* LFMB 19	6.8	6.2	Batch, flask	Waste gycerol	Yeast extract + (NH_4_)_2_SO_4_	5.0–6.0	28°C	72 h	27.7% PUFA	Chatzifragkou et al., 2011
*Yarrowia lipolytica* LGAM S(7)1	43.0	8.1	Continous, STR	Glycerol	Yeast extract + (NH_4_)_2_SO_4_	6.0	28°C	–	–	Papanikolaou and Aggelis, 2002
*Zygorhynchus moelleri* MUCL 1430	42.4	3.7	Batch, flask	Waste gycerol	Yeast extract + (NH_4_)_2_SO_4_	5.0–6.0	28°C	192 h	51.2% PUFA	Chatzifragkou et al., 2011
*Zygosaccharomyces rouxii* LFMB 3	12.5	5.5	Batch, flask	Waste gycerol	Yeast extract + (NH_4_)_2_SO_4_	5.0–6.0	28°C	168 h	15.4% PUFA	Chatzifragkou et al., 2011

a *Estimated from figure*.

The most frequently used carbon source is glucose (compare Table 2). Various mono- or disaccharides and carboxymethyl-cellulose (CMC) were tested as carbon source for *Mortierella isabellina* by Zeng et al. (2013). In this study cellular lipid contents above 60% were generated with xylose, glucose and fructose as substrates. CMC was a poor substrate, implying the absence of a cellulase system (Zeng et al., 2013). Cultivation of *Cunninghamella echinulata* and *M. isabellina* showed that the carbon source is a crucial parameter for the production of SCO as well as γ-linolenic acid (GLA, 18:3, ω-6). Both fungi showed satisfactory growth on glucose, fructose, and molasse, while *M. isabellina* failed to grow on saccharose (Chatzifragkou et al., 2010). Due to the high amounts of carbon source necessary to trigger the lipid accumulation it is economically effective to use low-cost raw materials, such as glycerol (Fakas et al., 2009b; Chatzifragkou et al., 2011; Tchakouteu et al., 2015), commercial sugars (Chatzifragkou et al., 2010), plant material (Lin et al., 2010; Economou et al., 2011b; Zeng et al., 2013; Matsakas et al., 2015), and lignocellulosic materials (Zeng et al., 2013). Glycerol, a waste product of the biodiesel production was tested for example by (Chatzifragkou et al., 2011) as a carbon source for 15 eukaryotic microorganisms. The tested yeasts accumulated up to 22% (w/w) lipids. On the contrary, the tested fungi showed cellular lipid contents of 18 to 43% (w/w). No difference between the oil accumulation of *C. echinulata* and *M. isabellina* were observed when either using raw glycerol or pure glycerol as carbon source. The successful application of plant wastes like tomato, potato or orange peels was shown by El-Fadaly et al. (2009), Gema et al. (2002), Zhao et al. (2010) resulting in cellular lipid concentrations above 50%.

Besides the selection of a suitable carbon source, the nitrogen source influences the accumulation of SCO. As well organic and inorganic nitrogen sources are used individually or in combination in the literature. These include yeast extract, urea, peptone, glycine, KNO_3_, NH_4_NO_3_, and (NH_4_)_2_SO_4_ (compare Table 2). Gao et al. (2013) investigated the influence of the nitrogen source when cultivating *M. isabellina* on xylose. The highest lipid accumulation (64.2%) was achieved with yeast extract. The influence of nitrogen compounds from tomato waste hydrolysate on the uptake of glucose was shown by Fakas et al. (2008). The removal of some quantities of organic nitrogen resulted in reduced glucose uptake and large amounts of biomass with low lipid content. The lipid accumulation was not effected when using glycerol as carbon source (Fakas et al., 2008). In addition to the selection of a suitable nitrogen source, the C/N ratio influences the lipid accumulation. Reported ratios range from 35 to 340 mol mol^−1^ (Papanikolaou et al., 2004; Fakas et al., 2009b; Ruan et al., 2012).

In principal, oleaginous microorganisms can be cultivated as batch, fed-batch or continuous cultures. Most of the reported experiments in literature are based on batch shaking flask cultivations (compare Table 2). A comparison of baffeled and unbaffeled flasks for *Schizochytrium* sp. showed that the use of baffled flaks increases the biomass production, the lipid accumulation and the concentration of docosahexaenoic acid (DHA, 22:6, ω-3; Ling et al., 2015). The cultivation of *M. alpina* in a stirred tank reactor resulted in an increase of lipid accumulated in the cells compared to shaking flasks (Stressler et al., 2013). In contrast, the lipid accumulation of *M. isabellina* was not affected by using a stirred tank reactor or shaking flasks, only the biomass production increased (Chatzifragkou et al., 2010).

An interesting feasibility study of an integrated process combining a heterotrophic cultivation of yeast with CO_2_ recycling in a phototrophic process was published by Dillschneider et al. (2014). The oleaginous yeast *Cryptococcus curvatus* and the oleaginous microalgae *Phaeodactylum tricornutum* were used and both processes revealed in lipid contents of about 40–45% (w/w).

### SSF for SCO production

SSF reproduces the natural microbiological processes such as food production, composting, and ensiling (Pandey et al., 1999). In general the advantages of SSF are a higher productivity, the possibility to use low cost media, reduced energy, and waste water costs. The disadvantages are for example difficulties in scale-up, in the control of process parameters and increasing cost for product recovery (Couto and Sanromán, 2006; Asadi et al., 2015).

An overview on SSF processes for the production of SCO is shown in Table 3. The amounts of produced SCO are given as weight percent based on the dried mixture consisting of substrate and biomass and are therefore difficult to compare. The achieved values range from 1.7 to 15.8% SCO per fermented mass. High amount of nutritionally valuable PUFA were produced in SSFs using *Mortierella* species (Fakas et al., 2009a; Stressler et al., 2013). In order to reduce costs, agro-industrial product, or residues such as wheat straw, cereal based products (bran, straw), wastes (orange peel, pear pomace, press cake, brewers spent grain) are used as substrates for SSF processes (Conti et al., 2001; Gema et al., 2002; Fakas et al., 2009a; Jacobs et al., 2010; Lin et al., 2010; Stressler et al., 2013). In dependence on the substrate and the enzymatic activity of the production strain a pretreatment of the substrate (chemically or enzymatically) is needed to break down insoluble material into available monomers (Peng and Chen, 2008). Lin et al. (2010) showed the direct microbial conversion of cellulose and wheat straw by *Aspergillus oryzae*. Furthermore, the substrate is often supplemented with nitrogen to obtain an ideal C/N ratio (Table 3).

**Table 3:** **SSF for the production of SCO: Species, lipid content, and cultivation conditions**.

**Species**	**SCO in fermented mass [% (w/w_DW_)]**	**Cultivation mode**	**Carbon source**	**Nitrogen source**	**pH [–]**	**Temperature [°C]**	**Duration**	**SCO Composition**	**References**
*Aspergillus oryzae* A-4	6.3	Batch, petri dishes	Wheat straw + wheat bran	(NH_4_)_2_SO_4_	–	30°C	360 h	–	Lin et al., 2010
*Cunninghamella elegans* CCF 1318	15.8	Batch, flaks	Barley	Yeast extract + peptone	–	28°C	420 h	–	Conti et al., 2001
	14.9	Batch, flaks	Millet	Yeast extract + peptone	–	28°C	420 h	–	Conti et al., 2001
	9.7	Batch, flaks	Wheat	Yeast extract + peptone	–	28°C	420 h	–	Conti et al., 2001
	13.8	Batch, flaks	Rice	Yeast extract + peptone	–	28°C	420 h	–	Conti et al., 2001
*Cunninghamella echinulata* ATHUM 4411	1.72	Batch, flaks	Orange peel		4.1–6.4	28°C	240 h	5.2% GLA	Gema et al., 2002
*Cunninghamella echinulata* ATHUM 4411	2.39	Batch, flaks	Orange peel + glucose	(NH_4_)_2_SO_4_	4.1–6.4	28°C	240 h	5.1% GLA	Gema et al., 2002
*Mortierella alpina*	16.4	Batch, tablet reactor	Oat bran		–	20°C	360 h	52% ARA, 74% PUFA	Stressler et al., 2013
*Mortierella isabellina* ATHUM 2935	12	Batch, petri dishes	Pear pomace		6.5	28°C	212 h	~27% PUFA	Fakas et al., 2009a
	9–11	Batch, petri dishes	Crusted sweet sorghum		6	28°C	192 h	–	Economou et al., 2010

### The genus *Mortierella* for PUFA production

As aforementioned, isolates of the genus *Mortierella* are excellent producers of SCO with high amounts of PUFAs (Bajpai et al., 1991; Stressler et al., 2013; Asadi et al., 2015). For the use in human nutrition PUFAs are of a special interest due to the high nutritional value (see Section Human Nutrition and Food Application). The genus *Mortierella* can be divided into two subgenera *Mortierella* (*M. alpina, M. hyalina, M. elongata*) and *Micromucor* (*M. ramanniana, M. isabellina, M. vinaces*), varying in their composition of SCO (Dyal and Narine, 2005). High amounts of ARA up to 70% are produced by *M. alpina*. The genera *M. hyalina* and *M. elongata* produce up to 23% ARA and tend to have higher concentrations of oleic acid (18:1; Dyal and Narine, 2005). Isolates belonging to the subgenera *Micromucor* accumulate high proportions of linoleic acid (LA; 18:2, ω-6) (up to 25%) and GLA (up to 31%). However, they tend to the production of high concentrations of oleic acid (Dyal and Narine, 2005). Strains of *M. alpina* were investigated by several researchers. Bajpai et al. (1991) achieved in a submerged fermentation of *M. alpina* ATCC 32222 9.6 g ARA per 100 g dried biomass (54% of total SCO) when using glucose and yeast extract as carbon and nitrogen source, respectively. The cultivation of *M. alpina* ATCC 32222 on soluble starch as carbon source ended up in 14.3 g ARA per 100 g dried biomass (66.4% of total PUFAs). Stressler et al. (2013) used an own isolated strain of *M. alpina* for the production of PUFAs by submerged fermentation. After 10 days of cultivation on glucose and yeast extract 10.3 g ARA per 100 g biodrymass (38% of SCO) were received.

### Microalgae as PUFA producers

Besides *Mortierella* species microalgae are also important producers of valuable PUFAs essential for human nutrition like EPA, DHA, and ARA (an overview is given by Yap and Chen, 2001; Huang et al., 2013). The most prominent DHA producer amongst microalgae is the heterotrophic dinoflaggelate *Crypthecodinium cohnii* containing more than 50% (w/w) DHA of total fatty acids (Jiang and Chen, 2000; Ratledge et al., 2001; de Swaaf et al., 2003a,b) and is also used commercially (DHASCO™; see Section Human Nutrition and Food Application). Other significant DHA producers are green microalgae of the genus *Schizochytrium*, e.g., *Schizochytrium* sp. S31 (Wu et al., 2005), *Schizochytrium* G13/2S (Ganuza and Izquierdo, 2007), and *Schizochytrium limacinum* (Chi et al., 2007).

An indisputable advantage of autotrophic microalgae is the ability to produce lipids from CO_2_ and sunlight. In non-sterile open pond systems microalgae can be cultivated in large scale under natural growth conditions and minimal costs for construction and operation. However, suboptimal cultivation conditions like low dissolved CO_2_ concentrations and inconsistent light intensities result in low cell densities making downstream processing cost-intensive. Furthermore, open pond systems are restricted to a limited number of species, either very robust e.g., toward high salinity or very fast-growing to be successful against competitive species (Yap and Chen, 2001; Ratledge and Cohen, 2008). Usually, bulk products for human diet supplement such as carotenoids or biomass, sold as powdered algae/cyanobacteria (e.g., *Spirulina*) are produced in open pond systems. However, high-value products like highly purified PUFAs for human nutrition can also be produced economically in well-controlled environments like photobioreactors. The high achievable prizes of the product justify higher production costs. In photobioreactor higher cell-densities can be reached while using significantly less space compared to open pond systems (Menetrez, 2012).

## Downstream processing

Downstream processing costs are one of the major obstacles to be solved for full economic efficiency of microbial lipids. Because single cell oils are formed intracellular for storage purposes, they have to be extracted upon further applications as long as production strains are not engineered to excrete TAGs or free fatty acids which would drastically simplify downstream processing. However, metabolic engineering efforts for secretion of TAGs from oleaginous strains have not been reported yet, but for engineered *Escherichia coli* (Lu et al., 2008; Lennen and Pfleger, 2012; Liu et al., 2012; Meng et al., 2013; Xu et al., 2013) and *Sacharomyces cerevisiae* (Michinaka et al., 2003; Leber et al., 2015) strains secreting free fatty acids.

For the extraction of lipids from microbial biomass cell disruption is most important, because efficiency of cell disruption directly influences subsequent downstream operations and overall extraction efficiencies (Senanayake and Fichtali, 2006). A multitude of cell disruption and lipid extraction methods are available which can be roughly divided in mechanical and non-mechanical methods. Nevertheless, depending on microorganism, scale, economics, and lipid application the method spectrum is narrowed to a few. Consequently, microbial lipids applied in food industry cannot be extracted with toxic solvents or should in the best case avoid any solvents to prevent solvent residues in food or contaminations with heavy metals (Uematsu et al., 2002; Sahena et al., 2009). Also, some methods may be highly suitable for analytical purposes but may not be applicable in industrial large scale operations due to high costs or simply a non-scalable extraction set-up. Additionally, the optimal method in terms of recovery can vary with each production strain and has to be elucidated for each strain separately as shown in comparative studies with microalgae (Lee et al., 2010; Li et al., 2014). Considering all limitations the optimal extraction method should enable a rapid, reproducible, quantitative, cost-effective, and non-toxic removal of lipids under mild conditions to prevent oxidative damage to polyunsaturated fatty acids.

Literature comparing systematically large scale cell disruption and extraction methods is scarce. Nevertheless, as microalgae oil production seems to receive more and more attention during the last years several recent comparative studies are now available dealing with downstream processing. For oleaginous filamentous fungi literature is even less found. Most of the studies are comparing different solvent extraction methods but pretreat the in liquid N_2_-frozen mycelium mechanically with mortar and pestle on bench scale which cannot be adapted to larger scales but is maybe somehow comparable to bead milling.

In the following an overview is given about common cell disruption and extraction methods (summarized also in Table 4).

**Table 4:** **Summary and comparison of different cell disruption and extraction methods**.

**Method**	**Advantages**	**Disadvantages**	**Scalability**	**Remarks**
**CELL DISRUPTION METHODS**				
**Mechanical methods**				Species independent, effective, no product contamination
Bead milling	Simple and efficient	Less efficient for bacteria	From lab to industrial scale	
Homogenization	Well-established in industry for other applications	Less suitable for filamentous fungi	To industrial scale	
Ultrasound	Continous operation possible	Heat generation and radical formation	Large scale not possible	
**Physical methods**				Limited scalability
Decompression	Gentle technique, minimizes chemical and physical stresses, and heat development	Less suitable for cell with tough cell wall, e.g., yeast, fungi and spores	Potentially larger scales	
Osmotic shock	Gentle technique, microorganims with cell walls are only weakened, not destroyed	High costs of additives	Smale scale only	
Microwaves	No drying necessary, quick, and inexpensive	Heat development, free radicals	Industrial scale for other applications	
Pulsed electrical field		Cell suspension has to be free of ions, cell disruption decreases gradually	Potentially larger scales	
Drying	Easily scalable	Energy demands depend on method, potentially very energy intensive, yeasts and plant cell only poorly affected	Industrial scale for other applications	Crucial for effective downstream processing, conservative effect
**Chemical methods**				Contamination of the products, unsuitable for some applications
Solvents	Possibly combines cell disruption and extraction	Cell walls of most microorganisms are usually impermeable to most solvents, large amounts of solvents necessary	Industrial scale	
*In situ* transesterifica-tion	Combines cell disruption, lipid extraction and transesterification	Chemical modification of the product –> suitable for analytical means or biodiesel production	Easily scalable	
**Enyzmes**	Mild reaction conditions, substrate specific, environmental friendly, safe for food applications	Specific enzyme cocktails needed for every microorganism, possible very expensive	Large scale application possible but dependent on enzyme costs	
**EXTRACTION METHODS**				
**Classical methods**	Established procedures	Requires high amounts on solvents	Analytical to industrial scale	
Soxhlet	automated systems available, combinable with other methods	requires a lot of time and high amounts of solvents, not suitable for thermosensitive compounds	Analytical to large scale	
Bligh and Dyer	Requires less solvents than Folch methods, also wet samples extractable	The unmodified method underestimates significantly lipid content for samples with < 2% lipid content	Analytical to large scale	
Folch	Standard technique for total lipid extraction, very liable	Needs dry samples, higher amounts of solvents than Bligh and Dyer	Analytical to large scale	
**Pressurized liquid extraction**	Enhanced extraction performance due to enhanced solubility and mass transfer properties, 5-fold faster and requires 20-fold less solvent than Bligh and Dyer, automated	High investment costs	Potentially large scale	
**Supercritical fluid extraction (CO_2_)**	Extraction can be performed at low temperatures, enabling a gentle extraction of thermosensitive compounds, protection against oxidation, environmental friendly	Moisture content of the sample hinders extraction efficiency, high investment costs	Industrial scale for other applications	

### Mechanical disruption methods

Cell disruption by mechanical methods are achieved by high stress to the cells due to cavitation, shear and/or impingement using high pressure, abrasion, or ultrasound resulting in non-specific cell wall breakdown (Chisti and Moo-Young, 1986; Harrison, 1991; Middelberg, 1995; Geciova et al., 2002). They are often combined with solvent extraction. In terms of cell disruption effectiveness, mechanical methods have great industrial potential as these methods seem to be less dependent on species (Klimek-Ochab et al., 2011; Lee et al., 2012) and many are applicable on industrial scale. Contamination with chemicals of the product lipid is unlikely as long as no chemicals are used for pretreatment. However, employing mechanical methods result in heat generation making cooling necessary in order to prevent damage to heat sensitive lipids (Geciova et al., 2002; Lee et al., 2010; Lee et al., 2012). Most of the currently used equipment was originally designed for other commercial purposes such as the homogenization and size reduction of paint and milk, but has been successfully adapted for cell disruption means (Chisti and Moo-Young, 1986). Oil recovery by mechanical pressing as it is commonly used for oilseeds like sunflower or rapeseed is not effective for microorganisms due to their small size enabling bypassing the press. Furthermore, the obligatory drying process prior to pressing requires a lot of energy (de Boer et al., 2012).

#### Bead milling

Cell disruption by bead milling is simple, effective, and suitable for a wide range of microorganisms. Cells are disintegrated by the impact of grinding beads and biomass as well as by compaction and shearing actions and the resulting energy transfer (Middelberg, 1995). Disruption efficiency is further depending on bead size and type, agitator velocity, flow rate, cell concentrations, bead loading, and microorganism (Chisti and Moo-Young, 1986; Middelberg, 1995; Doucha and Lívanský, 2008). Various types of bead mills are available both for lab-scale and industrial-scale. Effective cell disruption have been successfully demonstrated for yeast species, e.g., *S. cerevisiae, S. carlsbergensis, Candida boidinii* (Limon-Lason et al., 1979; Schütte et al., 1983; Hummel and Kula, 1989; van Gaver and Huyghebaert, 1991; Heim et al., 2007), algal species, e.g., *Chlorella* P12, *Botryococcus* sp., *Chlorella vulgaris, Scenedesmus* sp. (Doucha and Lívanský, 2008; Lee et al., 2010), fungal species, e.g., *Rhodotorula gracilis, Aspergillus fumigatus, Penicillium citrinum* (Klimek-Ochab et al., 2011), as well as for bacterial species, e.g., *Bacillus cereus, E. coli, Rhodococcus* sp. (Hummel and Kula, 1989). However, disruption efficiency for bacteria is considerably lower, due to the smaller cell dimension (Schütte et al., 1983). In terms of energy consumption, bead milling is more efficient, when high biomass concentrations are processed and the extracted products can be easily separated afterwards (Greenwell et al., 2010). To recover microbial oils, solvent extraction is generally performed subsequently to the bead milling process. Bead milling prior to solvent extraction has been shown to enhance oil recovery for the oleaginous yeast *C. curvatus* compared to treatment with microwaves or autoclaving. For the oleaginous fungus *M. isabellina* bead milling resulted in acceptable oil recovery but was surpassed by direct Soxhlet extraction of the mycelium (Yu et al., 2015).

#### Homogenization

Beside the original purpose of high-pressure homogenizers to mix, disperse, and reduce particle size in emulsions and suspensions, the adaption to cell disruption in biotechnology for the release of intracellular products has been successfully realized and can be applied on large scale. During the homogenization process, biomass is forced under high pressure through an orifice (Chisti and Moo-Young, 1986; Middelberg, 1995). The exact mechanisms and causes for cell disruption are still conversely discussed and were summarized by Clarke et al. (2010). Cell disruption efficiency is dependent on applied pressure, number of passes and organisms (Chisti and Moo-Young, 1986) and has been used successfully for yeast species, e.g., *S. cervisiae* (Spiden et al., 2013), algae species, e.g., *Chlorococcum* sp. (Halim et al., 2012), bacterial species, e.g., *E. coli* (Ling et al., 1997), and also some filamentous fungi, e.g., *Rhizopus nigricans* (Keshavarz et al., 1990). However, disruption of highly filamentous fungi by high-pressure homogenization have been found not very suitable due to blocking and clogging of the homogenizing valve by mycelium and fungal pellets (Chisti and Moo-Young, 1986; Hopkins, 1994; Middelberg, 1995) which can be prevented by using low biomass concentrations (Keshavarz et al., 1990). As a pretreatment for microbial lipid extraction high-pressure homogenization was applied to the oleaginous yeasts *C. curvatus* (Thiru et al., 2011) and *Pichia kudriavzevii* (Sankh et al., 2013), but was not compared to other pretreatment methods for its efficiency in SCO recovery. It has to be considered, however, that homogenization was originally applied to form emulsions. Therefore, the mixture of wet cells, SCOs and other intracellular lipids, especially phospholipids, is forming a very stable emulsion which is not easy to break complicating considerably lipid recovery.

#### Ultrasound

Ultrasound using frequencies around 25 kHz is another liquid-shear method which is frequently used in other industries, e.g., emulsification, degassing, or defoaming, and was found to be suitable for cell disruption. The most relevant effects of ultrasound on microorganisms are mechanical and can be attributed to cavitation, meaning the formation, growth, and collapse of gas bubbles. The collapse of gas bubbles results in shock waves creating liquid shear forces which disrupt cells (Chisti and Moo-Young, 1986; Thompson and Doraiswamy, 1999). However, the cavitation process generates high temperatures making on the one hand cooling necessary and on the other hand promotes radical formation enabling a wide range of chemical reactions with possible damage to the product (Suslick, 1989; Lee et al., 2012). Although it is possible to operate sonicators batch-wise or even continuously with minimal heat production (James et al., 1972; Borthwick et al., 2005), application for large-scale cell disruption is not feasible due to the difficulty of energy transmission to larger volumes (Chisti and Moo-Young, 1986). For an efficient cell disruption optimization of sonication time, cell density, power input, cycle number, and operation mode (batch-wise or continuous) has to be performed for the respective microorganisms. By optimizing sonication conditions an effective cell disruption was shown for the microalgae *Scenedesmus dimorphus* and *Nannochloropsis oculata* (McMillan et al., 2013; Wang and Yuan, 2015), the yeast *S. cerevisiae* (Jayakar and Singhal, 2012), the bacterium *E. coli* (Ho et al., 2006), and the filamentous fungi *P. citrinum* and *A. fumigatus* (Klimek-Ochab et al., 2011). However, due to the different definitions and measurements of the respective studies regarding cell disruption a general statement about the disruption suitability and efficiency of sonication is difficult. In some studies disruption efficiency is measured by the release of specific enzymes or proteins in general or other metabolites, in other studies it is evaluated by examination of the cells under the microscope as done by McMillan et al. (2013). An evaluation of cell morphology under the microscope might be better suited for a conclusion regarding cell disruption, but the release of metabolites or the activity of a specific enzyme might be better suited for the evaluation whether sonication is a suitable method for the extraction of the respective product. However, to enhance product release from a cell, a complete cell disruption might not be necessary. Permeabilisation of the cell in combination with another extraction step might be sufficient. In case of lipid extraction sonication is used in combination with solvent extraction and often called ultrasonic-assisted extraction. For *C. curvatus* ultrasonic assisted extraction of SCO has been shown to be beneficial in comparison to other methods, e.g., microwave-assisted, but for *M. isabellina* or *Chlorella sorokiniana* sonication proved not to be suitable (Yu et al., 2015). Ultrasonic pretreatment for lipid extraction can be problematic as it may affect the quality of lipids negatively for example by lipid oxidation of polyunsaturated fatty acids by free radicals (Gerde et al., 2012).

### Non-mechanical disruption methods

Cell disruption in a gentler way with the possibility of a more specific targeting of cell wall components can be achieved by non-mechanical methods. These methods require less energy but their application is often restricted to small scale processes due to limitations in process economy and efficiency (Klimek-Ochab et al., 2011).

#### Physical methods

Several physical disruption methods are available, including decompression, osmotic shock, microwave-treatment, pulsed electrical fields, and (freeze)-drying. However, applicability to process scale is limited for most of these methods.

Cell disruption by **decompression** is achieved by mixing cell suspension with pressurized supercritical gas and subsequent release of the pressure. The gas which has entered the cells expands upon pressure release and causes cell disruption due to the high pressure. It is a gentle technique, minimizing chemical and physical stresses as well as heat development and has the potential to be applied to larger scales (Middelberg, 1995; Simpson, 2010). Different gases can be used, e.g., nitrogen or carbon dioxide. Decompression with nitrogen has been shown effective for cell disruption of mammalian cells, plant cells, or bacteria, but less suitable for cell with tough cell wall, e.g., yeast, fungi, and spores (Simpson, 2010). For the disruption of wet yeast cells decompression with carbon dioxide was highly efficient, whereas under the same optimized conditions (pressure, temperature, and duration) dry cells were only poorly destroyed as well as when applying nitrogen for decompression. The low suitability of nitrogen was explained by its limited solubility in water (Nakamura et al., 1994). However, the inert gas nitrogen does not influence pH in contrast to carbon dioxide; furthermore, labile cell components are protected from oxidation (Simpson, 2010). Supercritical carbon dioxide can also be used for lipid extraction and will be discussed in Section Extraction Methods.

**Osmotic shock** is applied by exposing cells to a medium containing high concentration of a solute, e.g., salt or sugar exerting a high osmotic pressure and the subsequent sudden dilution of the medium resulting in an increase in intracellular pressure. Microorganisms with cell walls are not destroyed by osmotic shock, but weakened. Due to high costs of additives this methods is restricted to small scale applications (Middelberg, 1995).

**Microwaves** are oscillating non-ionizing electromagnetic waves with frequencies between 300 MHz and 300 GHz generating heat in dielectric or polar material by electric field-induced polarization and reorientation of molecules causing friction. The most effective range of dielectric heating are microwaves with frequencies between 915 MHz (for industrial application with greater penetration depth) and 2450 MHz (domestic microwave ovens and extraction applications; Leonelli and Mason, 2010; Routray and Orsat, 2012). The high cell disruption potential of microwaves is based on their interaction with the abundant free water molecules within cells resulting in localized heating, i.e., a sudden non-uniform temperature rise especially e.g., in vacuoles where free water is available in larger portions. Due to the volume expansion of the heated water intracellular pressure increases followed by spontaneously cell rupture (Chuanbin et al., 1998). The combination of microwave treatment with solvent extraction (microwave-assisted solvent extraction) has the potential of a quick and inexpensive method of lipid extraction as amounts of solvents can be reduced, drying of biomass prior to extraction is not necessary and yield can be increased compared to a simple thermal treatment (Cravotto et al., 2008; Balasubramanian et al., 2011; Mercer and Armenta, 2011; Rakesh et al., 2015). As microwave chemistry is already applied in many fields in industrial scale, e.g., drying, defrosting, food lyophilization, or devulcanization of rubber (Leonelli and Mason, 2010) application of microwave assisted solvent extraction of microbial lipids should be possible in large scales. However, similar to ultrasonic-assisted extraction, development of heat, and free-radicals as well as possible chemical conversions might damage polyunsaturated fatty acids influencing product quality (Yoshida et al., 1990; Günerken et al., 2015).

Cell disruption using a **pulsed electrical field** treatment is essentially accomplished by pore formation in cell membrane and cell wall as used for DNA transformation experiments via electroporation (Ho and Mittal, 1996). Besides cell destruction, pulsed electrical field treatment also leads to a temperature increase effecting and destroying intracellular molecules as well as an increase in lipid extraction (Sheng et al., 2011a; Zbinden et al., 2013; Lai et al., 2014). Pulsed electrical field treatment is promising in combination of extraction methods to recover lipids with the potential of large scale application (10,000 L/h capacity, Diversified Technologies, 2010) as well as continuous lipid extraction systems (Flisar et al., 2014). Like microwave treatment, wet cells can be used, however, cell suspension has to be free of ions, making washing steps, and/or deionization steps necessary. Additionally, cell disruption decreases gradually due to an increase of conductivity by the release of intracellular compounds (Günerken et al., 2015).

**Drying** of biomass is often done prior to other cell disruption and/or extraction techniques. In fact, most studies in this review use dry biomass for further downstream processing or analytics and drying is seen as crucial for effective downstream processing with very few exceptions. However, the drying methods (sun drying, oven drying, or freeze drying) had very little effect on the subsequent cell disruption technique when recovering lipid from *Scenedesmus* sp. as reported by Guldhe et al. (2014). In combination of thawing, drying itself can be a cell disruption method, though. During the process of slow freezing, large ice crystals are formed within the cell causing the rupture of intracellular membrane compartments. This method is inexpensive and easily scalable; however, plant cells need a large number of freeze-and-thaw-cycles, whereas yeast cells are only poorly affected (Hopkins, 1994).

#### Chemical methods

Cell disruption or permeabilization can be accomplished by a variety of chemicals, e.g., antibiotics, chelating agents, chaotropes, detergents, solvents, alkalis, and acids with different selectivity, efficiency and mode on action to the respective cell wall components of different microorganisms. Many chemical treatments are excluded in food applications or make at least an intensive downstream-processing necessary as the chemicals contaminate the products and are often of non-food grade (Middelberg, 1995; Geciova et al., 2002). Harsh chemical conditions may also damage the product.

Application of **solvents**, however, offers the possibility of combining cell disruption and lipid extraction without further pretreatment. Classical methods like Bligh and Dyer (1959) and Folch et al. (1957) can be used for wet and dry biomass but uses large amounts of organic hazardous solvents, like chloroform and methanol. Adaptations of these methods using less toxic solvents, like hexane/isopropanol, have been developed, though (Hara and Radin, 1978). Lipid extraction from microalgae using aqueous isopropanol (Yao et al., 2013) and ethanol (Yang et al., 2014) has been successfully shown. However, cell walls of most microorganisms are usually impermeable to most solvents (Sobus and Homlund, 1976; Jacob, 1992; Lee et al., 1998; Ryckebosch et al., 2012), therefore, at least a cell conditioning or pre-treatment has to be applied prior to solvent treatment to enhance solvent contact and extraction efficiency (Jacob, 1992). Different solvent extraction methods will be explained in detail in Section Extraction Methods.

**Acid catalyzed *in situ* transesterification** of either wet (Johnson and Wen, 2009; Cerón-García et al., 2013; Kim et al., 2015; Macías-Sánchez et al., 2015) or dry biomass (Liu and Zhao, 2007; Johnson and Wen, 2009; D'Oca et al., 2011; Li et al., 2011; Wahlen et al., 2011; Xu and Mi, 2011) combines cell disruption, lipid extraction, and transesterification to fatty acid methyl or ethyl esters (FAME or FAEE, respectively) for biodiesel production. Although studied extensively with oleaginous microalgae, *in situ* transesterification as also applied to oleaginous yeast, e.g., *Lipomyces starkeyi, Rhodosporidium toruloides* (Liu and Zhao, 2007), and fungi, e.g., *M. isabellina, Aspergillus candidus* (Liu and Zhao, 2007; Kakkad et al., 2015).

Already in 1985 Harrington and D'Arcy-Evans compared “conventional and *in situ* methods of transesterification” on sunflower seed oil. They concluded and were confirmed afterwards by several other studies dealing with oleaginous microorganisms (e.g., *Chlorella* species, Ehimen et al., 2010) that the fatty ester composition from *in situ* transesterification did not differ from that of pre-extracted oils reaching even higher yields when using the *in situ* method. However, when using H_2_SO_4_ as the acid catalyst moisture decreases heavily the yield of transesterification, making drying of biomass absolutely necessary (Harrington and D'Arcy-Evans, 1985; Ehimen et al., 2010). Drying, however, consumes a lot of energy and accounts considerably to the total production costs of low-value products like biodiesel (Molina Grima et al., 2003; Mata et al., 2010). Nevertheless, some studies showed, that by changing the catalyst to either HCl (Kim et al., 2015) or acetyl chloride (Macías-Sánchez et al., 2015) even wet biomass can be used for efficient direct *in situ* transesterification.

#### Use of enzymes

Cell disruption with lytic enzymes possess several advantages, e.g., mild reaction conditions and therefore prevention of shear and other stresses, substrate specificity, environmental friendly, and safe for food applications. Enzymes attack specifically cell wall components leading to a release of intracellular products. Due to their specificity, different enzymatic cocktails are needed for different microorganisms and the effectiveness of cell disruption is dependent on the enzyme type (Zheng et al., 2011). Enzymatic lysis as a tool for cell disruption has been extensively studied, especially for yeast and *E. coli* cells and several enzymes are available for all kinds of applications (reviewed by Salazar and Asenjo, 2007). The effectiveness of an enzymatic treatment in combination with solvent extraction, pressing, or ultrasound has been demonstrated for the lipid extraction of microalgae (Zheng et al., 2011) as well as for oilseeds (Shah et al., 2004; Soto et al., 2007) and the oleaginous yeast *R. toruloides* (Jin et al., 2012). Application on large scale is possible but depends heavily on the costs. When expensive enzyme cocktails are required for an effective cell destruction of certain organisms, enzyme immobilization may be a solution to lower enzyme costs due to the possibility of enzyme recycling. The feasibility of (re-)using immobilized cellulase to hydrolyze microalgae with subsequent lipid extraction was shown by Fu et al. (2010).

### Extraction methods

Lipid extraction (as every other extraction procedure) aims to separate efficiently and specifically the desired class of lipids from other cellular components, i.e., proteins, polysaccharides, non-desired lipids, and other small molecules. For an efficient and specific lipid extraction the characteristics of the particular microorganisms regarding cell wall structure and lipid content (sample matrix) have to be taken into account as well as the chemical structure, characteristics and location of the desired lipid (analyte; Mustafa and Turner, 2011). Furthermore, extracted lipids have to be preserved against oxidation and degradation by enzymes. Additionally, the future application of the lipids has to be considered when choosing extraction methods and solvents, especially when aiming for food applications, non-toxic and non-harmful solvents which are easily removable and recoverable are preferred.

The choice of solvents depends on the polarity of lipids to be extracted. Storage lipids, like triacylglycerols, are neutral lipids, and are therefore extracted with rather non-polar solvents, e.g., chloroform or hexane, whereas more polar lipids, like phospholipdis and glycolipids can be extracted with more polar solvents, e.g., alcohols. Mixtures of solvents, especially non-polar and polar systems, can enhance the extraction efficiency (Lee et al., 1998; Ryckebosch et al., 2012; Li et al., 2014; Ramluckan et al., 2014; Byreddy et al., 2015) due to the better release of lipids from protein-lipid complexes by the polar solvent and subsequent dissolving of the lipids in the non-polar solvent.

#### Classical extraction methods

The **Soxhlet Extraction** was invented by Franz Soxhlet in 1879 for the lipid extraction from milk powder (Soxhlet, 1879) and is one of the most common semi-continuous methods for lipid extraction from solid food samples. The sample is dried, ground to a fine powder and placed on a porous thimble inside the extraction chamber. The sample is extracted by several washing rounds with an organic solvent (originally petroleum ether) under reflux. After extraction, the solvent is evaporated and the residue is weighed, giving the total dry mass of extracted lipid. Several automated Soxhlet extraction systems were developed and are commercially available, e.g., Büchi extraction system B-811, however, the Soxhlet extraction is time consuming and requires high amounts of solvents. Nevertheless, in literature Soxhlet extraction is often used as standard method when comparing different extraction methods. It can be modified to enhance extraction convenience, for example by combining with microwaves (microwave-assisted Soxhlet extraction, García-Ayuso et al., 2000) which reduced considerably extraction time and solvent consumption. Soxhlet extraction is a highly useful extraction method for analytical purposes as it effectively extracts total lipids for class analysis. However, as stated by McNichol et al. (2012), it is not useful in extracting single lipid classes, e.g., TAG content for judging biodiesel potential, as it significantly overestimates the yield for total fatty acids due to the presence of several moderately polar lipid classes and other co-extracted compounds. Furthermore, Soxhlet extraction might not be suitable for extraction of lipids containing unsaturated fatty acids due to their instability at higher temperatures under reflux (Guckert et al., 1988).

The **Folch** extraction method (Folch et al., 1957) is generally accepted as a standard technique for recovering total lipids. It was originally invented as a simple method for the extraction of total lipids from animal tissues (brain, liver, and muscle) and uses the chloroform:methanol (2:1) solvent system and addition of salts to the crude extract. By washing the crude extract with water or a salt solution, a biphasic system is formed, with the lipid fraction in the lower phase and the non-lipid fraction in the upper (watery) phase. The Folch method is the most reliable extraction method for total lipids and is often used as a standard technique in extracting microbial lipids, similar to the Bligh and Dyer (1959) extraction method which is also used very often for this purpose. The difference between both methods is the ratio between the amounts of solvents and sample and the washing procedure. Originally developed as a fast and economic method to extract total lipids from large wet samples (frozen fish), it uses a minimal amount of chloroform:methanol (2:1) solvent mixture. However, in samples containing more than 2% of lipids, the Bligh and Dyer method underestimates significantly lipid content with raising inaccuracy with increasing lipid content (Iverson et al., 2001), therefore, it has to be modified when aiming to recover lipids from oleaginous microorganisms.

As stated above, other solvent systems have been evaluated in order to exchange toxic chloroform and methanol mixtures. Hexane is often applied as unpolar substitute and gives good results when extracting triacylglycerols. However, when aiming for total lipid extraction, other solvent systems than chloroform-methanol mixtures result in lower extraction efficiency, and are more sensitive to the water content of the sample (Fishwick and Wright, 1977; Hara and Radin, 1978; Guckert et al., 1988; Sheng et al., 2011b).

It has further been noted, that due to the high variation between organisms, pre-treatment, and extraction methods has to be adapted individually, resulting in a large variety of different procedures. Frequently, also new or simplified extraction methods are introduced in literature, (e.g., Ryckebosch et al., 2012; Axelsson and Gentili, 2014), however, these methods are mainly tested and applied to a very limited range of organisms or tissues, making comparability and transferability to other microorganisms very hard. As stated by Christie (1993) for consistent results with any method, it is inevitable to adopt a strict protocol following the principles of the original inventors.

#### Pressurized liquid extraction

Pressurized liquid extraction (PLE) is similar to Soxhlet extraction but uses liquid solvents at elevated temperatures and pressures resulting in an enhanced extraction performance due to enhanced solubility and mass transfer properties compared to methods at room temperature and without increased pressures (Richter et al., 1996, introduced as accelerated solvent extraction). As for Soxhlet extraction, solid or semi-solid samples are extracted but much less time and solvents are needed. Briefly, the sample is placed into an extraction cell which is heated to 80–200°C. The solvent is pumped into the extraction cell and remains a certain time, usually 5–10 min, under elevated pressure (10–20 MPa). Subsequently, fresh solvent is introduced and the extract is stored in a collection vial. Finally, the whole solvent is pushed out into the collection vial using pressurized nitrogen.

Originally introduced for the extraction of contaminants from diverse environmental samples and animal tissues, it is now applied for all kinds of bioactive compounds and nutraceuticals from wide varieties of samples, e.g., polyphenols, lignans, carotenoids, oils, and lipids, antioxidants etc. (see Mustafa and Turner, 2011 and citations within for a general overview).

In case of lipid extraction the efficiency as well as the effect of temperature and pressure on the extraction of polar and non-polar lipids from corn and oat was tested by Moreau et al. (2003). They revealed that for triacylglyceride extraction methylene chloride was best suited at an extraction temperature of 100°C. Microbial lipids have also been extracted successfully using PLE. In the studies of Macnaughton et al. (1997) and White et al. (2009) Phospholipids and neutral lipids were extracted as microbial lipid biomarkers as indicators for viable biomass and community structure of different environmental samples (soil, water, air). They concluded that PLE is more efficient in extracting some sorts of phospholipids from bacteria and fungi as well as in extracting eukaryotic neutral lipids, lipids from spores, and air-borne samples. PLE has also been applied in extracting total lipids from the oleaginous yeast *Rhodotorula glutinis* (Cescut et al., 2011). In their study, they optimized their PLE method using the response surface method by testing the influence of cycle duration, extraction temperature, and effect of dispersant. The optimized method was as efficient in extracting total lipids as the Bligh and Dyer method, but was 5-fold faster and required 20-fold less solvent with the advantage to be entirely automated. PLE equipment is commercially available (Dionex, ASE). Applying sequential PLE approaches, different lipid classes can be extracted separately. In a first step the sample is treated with n-hexane/acetone at 50°C to extract neutral lipids, and in a second round extraction is performed with chloroform/methanol at 110°C to obtain polar lipids. Combining sequential PLE with an “in-cell-fractionation” using silica-based sorbents, enables a highly efficient separation of neutral lipids and polar phospholipids (Poerschmann and Carlson, 2006).

#### Supercritical fluid extraction (SFE)

Supercritical fluids are defined as any substance above its critical temperature and pressure. In supercritical state, substances have highly desirable properties making them suitable for extractions: they can penetrate into and effuse through solids like a gas, but dissolve lipids or any other analyte like a fluid. By altering temperature and pressure above the critical point the density of the supercritical fluid and therefore the solubility of the analyte is enhanced. By adjusting temperature and pressure, the most suitable combination between penetration of the sample and solubility of the analyte can be achieved.

For extracting lipids or lipid-soluble compounds, supercritical CO_2_ is good solvent enabling a high level of recovery (Fattori et al., 1988). It also has several other advantages compared to organic solvents as it dissolves non-polar or slightly polar compounds, with a low solubility for water and no solubility of proteins, polysaccharides, sugar and salts (del Valle and Aguilera, 1989; Sahena et al., 2009). Due to the relatively low critical temperature of CO_2_ (31.1°C), extraction can be performed at low temperatures, enabling a gentle extraction of thermosensitive compounds, e.g., polyunsaturated fatty acids (Dron et al., 1997; Sahena et al., 2009). These compounds are also protected against oxidation due to the absence of oxygen during supercritical CO_2_ extraction (Bernardo-Gil et al., 2002). CO_2_ can be easily removed from the extract by simple decompression and can be recycled afterwards, therefore, it minimizes waste. Furthermore, it is non-toxic, non-flammable, non-polluting, inexpensive and inert, making it an ideal substance in food applications. Effectively, it is applied industrially in food processing, e.g., for decaffeination of coffee or tea and for several other applications (Raventós et al., 2002 and citations within). Using only CO_2_ as solvent in SFE, mostly non-polar lipids are extracted. If the extraction of more polar lipids or substances is desired, it can be achieved by addition of co-solvents like water, methanol or ethanol (Barth et al., 1995; Da Porto et al., 2014; Radzali et al., 2014). However, water content of the sample can negatively influences extraction efficiency, therefore, samples are usually dried or freeze-dried (Spanos et al., 1993; Berset et al., 1999) or have to be treated with a special surfactant (Walker et al., 1999).

Some studies also showed the suitability of SFE for microbial oil extraction, especially PUFAs, from oleaginous microorganisms. Walker et al. (1999) optimized a SFE method for lipid extraction of the filamentous fungus *Pythium irregulare* and showed the influence of moisture content and extraction efficiency on the solubility of lower- and higher molecular fatty acids. Certik and Horenitzky (1999) compared supercritical CO_2_ extraction of SCO containing GLA produced by *C. echinulata* with chloroform/methanol extraction according to Folch and a two-step Soxhlet extraction using hexane and ethanol in both lab-scale and semi-scale. They showed that although oil recovery by supercritical CO_2_ extraction was comparable to Soxhlet extraction, GLA content was about 10% higher than achieved with Soxhlet extraction. Furthermore, they revealed that oil extractability was mainly controlled by the oil solubility capacity of supercritical CO_2_ and is dependent on used CO_2_ volume. Sakaki et al. (1990) compared different supercritical fluids for the extraction of fungal SCO from *Mortierella ramanniana* var. *angulispora*. Oil solubility was found to be best in supercritical N_2_O followed by supercritical CO_2_ at different tested temperatures and pressures. Although both N_2_O and CO_2_ possess similar physical properties, e.g., molecular weight, critical temperature and pressure, oil solubility in supercritical N_2_O was nearly five times higher than in supercritical CO_2_. However, calculations for oil solubility in supercritical CO_2_ from Certik and Horenitzky (1999) were two times higher than from Sakaki et al. (1990) apparently due to different sample preparation and extraction conditions. In the study of Couto et al. (2010) supercritical CO_2_ extraction was found highly suitable for the extraction of lipids from the heterotrophic microalga *C. cohnii* which is rich in DHA. While the extraction according to Bligh and Dyer yielded in a mixture of lipid compound with different polarities, supercritical CO_2_ extraction was more selective and enriched PUFAs resulting in an extract containing 72% of DHA.

## Human nutrition and food application

Polyunsaturated fatty acids (PUFAs) affect many different physiological functions in the human body and are therefore important for the human health (Dyal and Narine, 2005; Bellou et al., 2016). PUFAs of the ω-3 and ω-6 class [e.g., α-linolenic acid (ALA; 18:3, ω-3), linoleic acid (LA; 18:2, ω-3)] play important roles not only as structural components of membrane phospholipids but also as precursors of the eicosanoids, which are essential for all mammals (Sakuradani et al., 2009; Stressler et al., 2013). Eicosanoids are hormone-like substances and influence the cardiovascular system, the immune system, the central nervous system, the brain and are involved by inflammatory reactions (Shinmen et al., 1989; Ward and Singh, 2005b; Arjuna, 2014; Bellou et al., 2016). In addition, for example, the cerebral cortex as well as the retina have a high amount of PUFAs such as arachidonic acid (ARA; 20:4, ω-6) and docosahexaenoic acid (DHA; 22:6, ω-3; Fontani et al., 2005; Ward and Singh, 2005b; Bellou et al., 2016). Furthermore, PUFAs are components of thrombocytes, neuronal and muscle cells as well as immunocompetent cells such as neutrophils and monocytes (Simopoulos, 1999; Ward and Singh, 2005b). Because mammals lack the ability to synthesize essential fatty acids (LA, ALA), these must be supplied by the diet (Laoteng and Certik, 2010). In humans these fatty acids can be converted to higher PUFAs, but this conversion often occurs with extremely low rates and therefore an external PUFA uptake is necessary (Bellou et al., 2016). It is postulated, that a balance in the uptake of ω-3 and ω-6 fatty acids in a ratio of 1:4 is important for the health and physical wellbeing of humans (Gill and Valivety, 1997; Simopoulos, 1999). In the food industry, lipids rich in PUFAs are highly demanded and used as food additives in order to enhance and/or supplement the fatty acid composition of specific foods such as infant food (Bellou et al., 2016). It is to be mention, that using fish oils as supplements is discussed critically, expressly for infants, due to the presence of environmental pollutants like dioxins, heavy metals and polychlorinated biphenyls (PCBs; Béligon et al., 2016). These components can be taken up from the fish oils and concentrated in the liver and other organs (Béligon et al., 2016). On the other hand, DHA and ARA are essential for the neural development and visual acuity of new-borns. Normally, the infants took up these PUFAs by the mother's milk (Granot et al., 2016). But if the mother's milk is replaced by cow's milk, the new-borns have a deficiency of these PUFA caused by their absent in cow's milk. Thus, ARA and DHA have to be added to the diet of infants to ensure a normal development (Béligon et al., 2016). In general, the enrichment of food with PUFAs can be realized by different ways: (i) The direct addition of PUFAs into food, (ii) the supplementation of food with PUFA-producing edible microorganisms, and (iii) the usage of animal feeds, rich in PUFAs, which results in animal products (e.g., eggs, meat) with increased PUFA contents (Bellou et al., 2016). Specially, microbial lipids, which are rich in PUFAs, can be extracted and added to various foods as pure oil or as stable emulsions (Bellou et al., 2016). As an alternative, some agricultural products (e.g., cereals) and by-products (e.g., orange peels, apple or pear pomace, sweet sorghum, etc.) can be enriched in PUFAs through solid or semi-solid state fermentation with PUFA producing microorganisms and directly used as food and/or feed supplements (Gema et al., 2002; Fakas et al., 2009a; Economou et al., 2010, 2011b; Čertík et al., 2013; Bača et al., 2014; Bellou et al., 2016).

The first commercial microbial oil was produced in 1985 and was rich in γ-linolenic acid (GLA; 18:3, ω-6). The oil was aimed at being an alternative source of the oil from seeds of evening primrose (*Oenothera biennis*; Wynn and Ratledge, 2005). The oil was produced by *Mucor circinelloides*, also known as *Mucor javanicus* and sold under the trade name of “Oil of Javanicus” (Wynn and Ratledge, 2005). Previous studies already showed that GLA exists in most, if not all, species in the group of *Mucorales* (Shaw, 1965, 1966; Wynn and Ratledge, 2005). However, in 1990 the production of Oil of Javanicus was folded because it could no longer compete against borage oil (*Borago officinalis*), which was developed during the second half of the 1980's, sold under the name “starflower oil” and had an even higher content of GLA than oil of Javanicus (Wynn and Ratledge, 2005).

In the mid of the 1960's an oil rich in ARA from a microbial source was investigated. The initial interest on this fatty acid was the misapprehension that it has the potential as chicken flavor additive (Wynn and Ratledge, 2005). A number of potentially useful organisms were identified by the research group of Bob Shaw at Unilever Ltd., (Shaw, 1965, 1966; Wynn and Ratledge, 2005). Beside the potential application of such an oil rich in ARA for the food industry, a non-dietetic application was as a cosmetic additive. The Lion Corp of Japan developed and patenting a process in 1988 for the production of ARA-rich oil (Wynn and Ratledge, 2005). In respect for a commercial production of ARA-rich single cell oil, only two fungi *Mortierella alpina* and *Pythium* sp. seem to be preferable (Kyle, 1997; Wynn and Ratledge, 2005). The following developments focused on *M. alpina* because it was considered as the most productive species (Shinmen et al., 1989; Stredanská and Šajbidor, 1992) and all current commercial processes for ARA-rich single cell oil used this fungus (Wynn and Ratledge, 2005). According to Wynn and Ratledge (2005) there are currently at least three commercial processes to produce ARA-rich single cell oils. Two of them are operating in the Far East (Suntory Corp., Japan; Wuhan Alking Bioengineering Co. Ltd., China) and the ARA-rich single cell oils are used for infant formula supplementation and as health supplements. But both processes do not constitute as a major global source for ARA-rich single cell oil. The largest proportion (>95% in 2005; Wynn and Ratledge, 2005) of the global ARA-rich single cell oil is produced by DSM Co. in Italy under contract for Martek Biosciences Corp. The DSM product is sold under the trade name ARASCO™ as part of the infant formula additive Formulaid™.

As mentioned above, DHA is important for infants to ensure a normal development and fish oil has significant quantities of it. However, due to the presence of environmental pollutants, the inclusion of fish oil into infant milk formula is not recommended (Béligon et al., 2016). Two heterotrophic microalgae, a dinoflagellate (*C. cohnii*), and a stramenopile (*Schizochytrium*) have been selected as production organisms for commercial DHA production (Wynn and Ratledge, 2005). *C. cohnii* has a total fatty acid content up to 50% and DHA corresponds to 95% of all PUFAs (Wynn and Ratledge, 2005). In *Schizochytrium* a significant amount (approximately one third of DHA) of docosapentaenoic acid (DPA; 22:5, ω-6) is also present and thus the appearance of this fatty acid in humans was associated with a deficiency of DHA, it was thought that this fatty acid should be avoided in infant formula (Wynn and Ratledge, 2005). In summary, while the *C. cohnii* oil was developed as an infant formula additive, the *Schizochytrium* oil was developed as a general food additive and as an animal food ingredient aimed at increasing the amount of DHA in the human diet in the form of meat and eggs (Barclay, 1991; Wynn and Ratledge, 2005). Since April 2002, Martek and OmegaTech have been a single company and both DHA-rich oils remain under development (Wynn and Ratledge, 2005). The product derived from *C. cohnii* is sold under the trade name DHASCO™.

From a commercial point of view, single cell oil became successful in the twenty-first century although the first SCO was launched on the market in 1985. The commercial breakthrough was based on the supplementation of infant formula with a mixture of ARASCO™ and DHASCO™ in many countries in Europe, Australia, and the Far East (Wynn and Ratledge, 2005). In May 2001, also the Food and Drug Administration (FDA) gave the GRAS status for DHA and ARA single cell oil for its inclusion in infant formula in the USA. Since the launch of single cell oil containing formula in February 2002 in the US market, the single cell oil fortified formula have captured over 50% of the US formula sales (Wynn and Ratledge, 2005). Since 1994 Martek's oils (also called life'sDHA™) have been used in infant formula and until 2010 it has been estimated that over 24 million babies, including more than 500000 preterm infants, have been fed infant formulas containing life'sDHA™ (Fichtali and Senanayake, 2010). Until 2010, life'sDHA™ oils are licensed and sold to 24 infant formula manufacturers, which represent more than 70% of the worldwide wholesale infant formula market (Table 5; Fichtali and Senanayake, 2010).

**Table 5:** **List of companies that produce DHA-supplemented infant formula (modified Fichtali and Senanayake, 2010)**.

**Company**	**Country**
Abbott Laboratories	USA
Aspen Pharmacare	South Africa
Heinz Wattie's Limited – a subsidiary of the H.J. Heinz Company	New Zealand
Royal Numico	The Netherlands
Laboratotios Ordesa	Spain
Materna Ltd	Israel
Mead Johnson Nutritionals – Bristol-Myers Squibb	USA
Medici Medical	Italy
Nestle	Switzerland
Nutrition and Sante Iberia, S.L.	Spain
Pasteur Milk	South Korea
PT Sanghiang Perkasa	Indonesia
Semper AB	Sweden
Synutra, Inc.	China
Wyeth Ayerst	USA
PBM Products, LLC	USA
Arla Foods	Denmark
Murray Goulburn	Australia
Namyang Dairy Products Co., Ltd	South Korea
Parmalat Colombia	Colombia
Hain Celestial Group	USA
Alter Farmacia	Spain
Nature's One	USA
Earth's Best	USA
Vermont Organics	USA

Before microbial single cell oil was used for infant formula, it was first explored in the 1980's for preparation of cocoa butter substitutes since cocoa butter was in short supply (Ward and Singh, 2005a). Therefore, *C. curvatus* was mutated to partially block the Δ-9 desaturase, which converts stearate (18:0) to oleate (18:1, ω-9), to increase the amount of stearate at the expense of oleate (Ward and Singh, 2005a). The oil obtained contained the fatty acids palmitic acid (16:0), stearic acid, and oleic acid in a ratio of 24:21:30 (%, w/w), which was quite similar to cocoa butter (28:35:35; %, w/w; Moreton, 1988; Verwoert et al., 1989; Davies, 1992; Ratledge, 2001; Ward and Singh, 2005a). However, the process became uncompetitive when the world price of cocoa butter dropped from $8000/ton to < $2500/ton (Ratledge, 2001; Ward and Singh, 2005a; Wynn and Ratledge, 2005).

Besides the infant formula, also commercial dairy products such as liquid milk and yogurt are currently fortified with ω-3 PUFAs obtained from flaxseed, fish oil, or even marine microalgae (Ganesan et al., 2014). In addition, vegetable and marine microalgae oils have been used to supply substantial amounts of ω-3 PUFAs in order to produce ω-3 PUFA-enriched meat products (Jiménez-Colmenero, 2007). Examples for products formulated with non-meat fats from algal are ground turkey patties, fresh pork sausage, and restricted hams (Lee et al., 2006a,b). Here, the oils were incorporated as oil-in-water emulsions with whey protein isolates as protein source. The advantage of such an emulsion is, that it is stable against oxidation and physically stable at pH 3.0 (Djordjevic et al., 2004). In general, unsaturated fatty acids are unstable and will oxidize quickly leading to formation of unpleasant smelling and tasting aldehydes and ketons (Kralovec et al., 2012). Various types of antioxidants have been used for the chemical stabilization of e.g., DHA, but these are not adequate to enable sensory stabilization of such oils for addition to many foods and beverages (Kralovec et al., 2012). In previously published articles, the usage of antioxidants to improve the stability of oils containing unsaturated fatty acids, were discussed (Shahidi and Zhong, 2010a,b). Beside the previously named emulsification, also microencapsualtion can improve the oxidative stability of fatty acids (Kralovec et al., 2012). Microencapsulation is a unique process that has been used not only to “convert” liquids to solids, but also to add functionalities or even to improve oxidative stability to ingredients (Kralovec et al., 2012).

One of the most important aspects of the design of potential functional foods is the scale of the alterations needed to achieve potential health-promoting functions. One of the possible limitations affecting fortified products is that large quantities may need to be consumed to assure recommended intake levels (Garg et al., 2006; Jiménez-Colmenero, 2007). For example, as mentioned above, non-meat fat (algal oil) was used to improve the lipid composition of various meat products. The effort required to replace animal fat varies widely depending on the fat content of the meat product. In products containing low fat (< 10%), regardless of the percentage of fat replaced, the presence of non-meat fat in the product is limited (≤ 5 g/100 g). In medium- to high-fat products, more animal fat is replaced and hence there is a larger intake of healthier non-meat lipids when the new product is consumed in its normal form (Jiménez-Colmenero, 2007). In general, one fundamental requirement of the design and reformulation of products with a view to potential health benefits is to assure that the lipid content and profile are optimum. The final product should contain enough of these beneficial compounds so that the quantity of the product that a person can reasonably be expected to consume supplies enough of the nutrient to produce the nutritional or physiological effect claimed on the basis of generally accepted scientific data (Jiménez-Colmenero, 2007).

## Other applications of microbial oils

Besides the well-established application of microbial oils as food supplements, other applications are thinkable and desired for a successful establishment of a more sustainable or biobased economy.

### Biodiesel

The idea of using SCO for the production of fatty acid methyl or ethyl esters, also known as biodiesel seems tempting and offers several advantages compared to biodiesel produced from plant or animal lipids. Fuel demands cannot be satisfied by using plant oil or animal lipids due to the large areas of production land required and the competition to food production (IEA, 2006). In contrast, microbial oil production is characterized by the short life cycle of microbes and the possibility of a production process not influenced by external factors such as venue, season, or climate (Thiru et al., 2011). The production of single cell oil in large scale is suited to avoid the conflict with plants used for energy and material industry, and to avoid the appearance of mono cultures and exhaustion of the soils. Furthermore, less land is needed for microbial production than for conventional agricultural production (Ratledge and Cohen, 2008). The production of biodiesel and its advantages and shortcomings has been exhaustingly reviewed in the last years (e.g., Ma and Hanna, 1999; Molina Grima et al., 2003; Chisti, 2007; Li Y. et al., 2008; Meng et al., 2009; Brennan and Owende, 2010; Mata et al., 2010; Liang and Jiang, 2013) with different focus on diverse aspects. However, the general conclusion of all authors is that the high costs of using SCO for biodiesel production hinder the commercial production. Unsurprisingly, the additional efforts needed for microbial oil production compared to biodiesel production from plant oil (including production in a bioreactor, substrate costs, and downstream processing as reviewed in this article) are responsible for the higher costs of a very cheap product. In the opinion of the authors biodiesel production from SCO will not be economically competitive as long as cheap fossil alternatives are available.

### Chemicals (“oleochemicals”)

Oleochemicals are usually defined as chemical products derived from plant or animal triacylglycerols (Rupilius and Ahmad, 2006). However, in the opinion of the authors, also microbial sources should be included to the definition. Basic oleochemicals include fatty acids, fatty alcohols, and methyl ester. Especially fatty alcohols and ester between a fatty acid and a fatty alcohol (wax ester) can be applied in various industries, e.g., in soaps, detergents, cosmetic additives, pheromones, and flavors (Noweck and Grafahrend, 2006; Steen et al., 2010). Fatty alcohols and wax ester are obtained either from natural source, i.e., plant and animal oil, by hydrogenation of triacylglycerides or directly from jojoba plant oil which consists of wax esters. Alternatively, the chemical synthesis using petrochemical feedstocks can also be used (Noweck and Grafahrend, 2006). According to Steen et al. (2010) the market value for fatty alcohols, aldehydes, and wax esters was ~3 billion $ in 2004 with a prize of 1500 $/per ton. The microbial production of tailor-made fatty acid derivatives offers therefore a huge market and an economic alternative for the production of biodiesel. The principal feasibility of this approach was demonstrated by Steen et al. (2010) and Youngquist et al. (2013). Using metabolic engineering of the non-oleaginous organism *E. coli* fatty alcohols and wax esters were produced from sugar. The microbial production of oleochemicals is also subject of several patents, e.g., Schirmer et al. (2012) (US 8,268,599 B2), Hu (2011) (EP 2 367 947 B1), and Osterhout and Burgard (2014) (US20140127765 A1).

## Conclusions and outlook

SCOs have been proven to be a highly interesting class of biotechnological products ranging from bulk chemicals to high-value products. Especially the occurrence of PUFAs in SCO similar as in plant oils classifies them to be very valuable resources with the advantages of short cultivation times of the producing oleaginous microorganisms and high product purity. However, major obstacles are still the extraction of the intracellular SCOs being difficult and cost-intensive. Therefore, commercialization has only occurred for high-value products, i.e., PUFAs in food applications. A general transfer of established oil extraction methods for plant oils is usually not applicable for oleaginous microorganisms due to the small size and the risk of emulsification. A step toward better profitability might be the realization of high cell densities achieved in short time, product purity, i.e., the enrichment of single desired fatty acids in SCO achieved by metabolic engineering, and the development of an universal and cost-efficient method for SCO recovery.

## Author contributions

KO is corresponding author, and has written Sections Introduction, Downstream Processing, and Other Applications of Microbial Oils of the manuscript. CG was responsible for literature aquisition and has written Sections Microorganisms for SCO Production and Processes for the Microbial Production of SCO of the manuscript. TS has written Section Human Nutrition and Food Application of the manuscript. LF and CS have been responsible for critical review of the manuscript as well as discussion and overall improvement. Together, they have written the Section Conclusion and Outlook.

### Conflict of interest statement

The authors declare that the research was conducted in the absence of any commercial or financial relationships that could be construed as a potential conflict of interest.
